# Effect of Gender to Fat Deposition in Yaks Based on Transcriptomic and Metabolomics Analysis

**DOI:** 10.3389/fcell.2021.653188

**Published:** 2021-08-24

**Authors:** Lin Xiong, Jie Pei, Xiaoyun Wu, Qudratullah Kalwar, Ping Yan, Xian Guo

**Affiliations:** ^1^Animal Science Department, Lanzhou Institute of Husbandry and Pharmaceutical Sciences, Chinese Academy of Agricultural Sciences, Lanzhou, China; ^2^Key Laboratory for Yak Genetics, Breeding, and Reproduction Engineering of Gansu Province, Lanzhou, China; ^3^Department of Animal Reproduction, Shaheed Benazir Bhutto University of Veterinary and Animal Sciences, Sakrand, Pakistan

**Keywords:** yak, fat deposition, transcriptome, metabolomics, PPAR signal, gender

## Abstract

Fat deposition in yaks plays an important part in survival, multiplication, and meat quality. In this work, the characteristic of fat deposition in male yaks (MYs) and female yaks (FYs) and the regulations of gender to yak fat deposition were explored by mRNA-Seq and non-targeted metabolomics analyses. FYs possessed a higher body fat rate (BFR) of visceral fat, fat content in longissimus dorsi (LD) and liver, and subcutaneous fat thickness (*p* < 0.05). The fat and cholesterol synthesis in liver and the fat transport in FY blood increased. The fat metabolism in yaks is the combined effect of carbohydrate, fatty acid, and amino acid metabolism by tricarboxylic acid (TCA) cycle, and an increase of triglyceride (TG) synthesis was accompanied by an increase of steroid synthesis. The high levels of myo-inositol and cortisol (COR) (*p* < 0.01) activated the calcium signaling in FY subcutaneous fat, followed by the increase of adipocyte secretion, and resulted in more leptin (LEP) secretion (*p* < 0.01). Then peroxisome proliferator-activated receptor (PPAR) signaling was activated by the focal adhesions and ECM–receptor interaction. Finally, the TG and steroid synthesis increased by the expression regulation of *ME1*, *SCD*, *ELOVL6*, *DGAT2*, *DBI*, *LPL*, *CPT1*, *PLIN1*, *LIPA, DHCR24*, and *SQLE* gene. The above genes can be considered as the candidate genes for yak with higher fat amount in molecular breeding in the future. This study can provide a theoretical basis for improving the meat quality and breeding of yaks.

## Introduction

Fat is very important to livestock and plays a key role in energy metabolism and animal product quality (Sadkowski et al., 2014; Gomez et al., 2018; Guo et al., 2018; Mason et al., 2019; Silva et al., 2019; Albuquerque et al., 2020). Yak (*Bos grunniens*) is a unique livestock in the Tibetan Plateau and its adjacent areas (Zou et al., 2019). As a classic grazing livestock, yaks suffer long-term starvation during the cold season and maintain life of its own by fat mobilization. The pregnancy and parturition of female yaks (FYs) largely depend on fat storage. Moreover, yak meat is the major animal protein in local human diet (Guo et al., 2014; Wen et al., 2015). Low fat content in yak meat leads to bad tenderness, flavor, and processability.

Heredity, diet, gender, and breed can affect the fat deposition in livestock (Baik et al., 2014; Gui et al., 2017). Livestock possessing different genders have noticeable alterations in fatty acid synthesis and fat deposition in adipose tissue (Moibi et al., 2000). Gender influences the metabolic responses to energy intake, and the effect of gender to fat deposition in livestock is realized by hormone regulation (Carr et al., 2001; Bracht et al., 2020; Bucław et al., 2020). A previous study reported that castration increases intramuscular adipogenesis, lipogenesis, and lipolysis (Jeong et al., 2013). There are very few reports on yak fat at present, and the study on the differences of fat deposition in FYs and male yaks (MYs) can improve yak meat quality and promote yak breeding and genetics. Fat deposition is a complex process (Cui et al., 2019), and it is difficult to expound it by a single technique. Transcriptome enables us to simultaneously and globally examine the complete responses at a transcriptional level (McNamara et al., 2016), and the newest RNA-sequencing (RNA-Seq) analysis can interrogate the mRNA level in yak adipose tissue to estimate its gene expression profile under different genders. Meanwhile, non-targeted metabolomics technologies can identify metabolic networks. The combined metabolomics and transcriptomics approach can reveal the differences of fat metabolism based on gene actions and metabolites in FY and MY adipose tissue.

In this work, a study was carried out to observe the characteristics of fat deposition in MYs and FYs and explore the effect of gender to fat deposition in yaks by transcriptomic and metabolomic analysis. Differentially expressed genes (DEGs) and differential metabolites (DMs) in FYs and FY subcutaneous fat were identified by mRNA-Seq and UHPLC-TOP-MS non-targeted metabolomics analyses and annotated by gene ontology (GO) and Kyoto Encyclopedia of Genes and Genome (KEGG) enrichment analysis. Basing on the above results, the important candidate genes and signaling pathways affecting fat deposition in yaks with different genders were identified and clarified. Moreover, additional investigations including enzyme-linked immunosorbent assay (ELISA), reverse transcription-quantitative PCR (qPCR), liquid chromatography–parallel reaction monitoring–mass spectrometry (LC-PRM-MS), and gas chromatography (GC) were performed to support or supplement above results. This research can establish a theoretical basis for the improvement of the yak meat quality and molecular breeding and promote the development of yak breeding.

## Materials and Methods

### Animal and Sample Collection

The animal experiment was carried out at a pasture in Haiyan County in Qinghai Province, China. FYs (*n* = 10, 4 years old) and MYs (*n* = 10, 4 years old) were kept in natural grazing mode and given free-choice access to diet and water. In late September, the body weights of MYs and FYs were 320 ± 10 and 260 ± 10 kg, respectively. Twenty milliliters of blood was collected from the jugular vein of each yak under limosis, and the serum samples were obtained by centrifugation at 4,000 r/min for 10 min. All yaks were sacrificed through electrical stunning, and the longissimus dorsi (LD, 12–13th rib level), liver, and subcutaneous fat were collected and kept in liquid nitrogen. The liver weights of MYs and FYs were 6.85 ± 0.48 and 5.62 ± 0.53 kg, respectively.

### Measurement of Fat Amount

Subcutaneous fat, perirenal fat, omentum majus, mesentery fat, fat around liver, LD fat contents, and liver fat content in 10 FYs and 10 MYs were measured. Subcutaneous fat thickness on back (on both sides of midline of the dorsal at the 5th–6th thoracic vertebra) and on waist (on both sides of midline at the cruciate region) were measured using a vernier caliper (Hengliang Inc., Shanghai, China). Body fat rates (BFRs) of perirenal fat, omentum majus, mesentery fat, and fat around liver were evaluated by the weighing method. The LD and liver fat contents were determined with a Soxtec 2050 Soxhlet apparatus (FOSS Inc., Hillerød, Denmark).

### Determination of Metabolite Concentrations in Serum and Enzyme Concentrations in Liver

The concentrations of metabolites involved in fat metabolism in serum and enzymes involved in fat metabolism in the liver of 10 FYs and 10 MYs were measured. Glucose (GLU), cholesterol (CH), TG, high-density lipoprotein cholesterol (HDL), low-density lipoprotein cholesterol (LDL), non-esterified fatty acid (NEFA), total protein (TP), and albumin (ALB) in serum were determined with a BS-420 automatic biochemical analyzer (Mindray Inc., Shenzhen, China). Very low-density lipoprotein (VLDL), leptin (LEP), insulin-like growth factor-1 (IGF-1), cortisol (COR), fatty acid synthase (FAS), acetyl-CoA carboxylase (ACC), stearoyl-CoA desaturase (SCD), diacylglycerol acyltransferase 1 (DGAT-1), hormone-sensitive lipase (HSL), lipoprotein lipase (LIPL), adipose triglyceride lipase (ATGL), carnitine palmitoyltransferase 1 (CPT-1) were measured with commercial bovine ELISA kits from Shanghai Xuanya Biotechnology Co., Ltd. (Shanghai, China). Alanine aminotransferase (ALT) and aspartate aminotransferase (AST) were measured with commercial bovine ELISA kits from Shuangyin Biotechnology Co., Ltd. (Shanghai, China).

### RNA Extraction and Sequencing in Subcutaneous Fat

Three subcutaneous fat samples were randomly selected in the FY and MY groups, respectively. Total RNA was extracted using the mirVana^TM^ miRNA Isolation Kit (Ambion Inc., Foster City, CA, United States) according to instructions. The mRNA libraries for sequencing were prepared with TruSeq Stranded mRNA LT Sample Prep Kit (Illumina, San Diego, CA, United States). Finally, these libraries were sequenced on the Illumina sequencing platform (HiSeq^TM^ 2500), and 125-bp paired-end reads were generated.

### Metabolite Extraction and MS Data in Subcutaneous Fat

Ten subcutaneous fat samples in the FY and MY groups were used in metabolomics analysis. Thirty milligrams of fat was extracted with the solution of 2-chloro-l-phenylalanine in methanol and methanol–water (4:1, v:v). After centrifugation at 13,000 r/min for 10 min, 300 μl supernatant was dried in a freeze concentration centrifugal dryer. The residue was dissolved in 400 μl solution of methanol–water (1:4, v:v), followed by being vortexed for 30 s and centrifuged at 13,000 r/min for 10 min. One hundred fifty microliters of supernatant was collected and filtered through 0.22-μm microfilters and then analyzed by UHPLC-TOP-MS with ACQUITY UPLC BEH C_18_ column (100 mm × 2.1 mm, 1.7 μm). The elution solution consisted of (A) water containing 0.1% formic acid (v:v) and (B) the mixed solution of acetonitrile and methanol (2:3, v:v) containing 0.1% formic acid. The elution program was as follows: 5–20% B over 0–2 min, 20–25% B over 2–4 min, 25–60% B over 4–9 min, 60–100% B over 9–17 min, holding at 100% B for 2 min, 100 to 5% B over 19–19.1 min, and holding at 5% B from 19.1 to 20.1 min. The flow rate, column temperature, and injection volume were 0.4 ml/min, 45°C, and 5 μl, respectively. The MS system was operated using the ESI+ and ESI− mode. The parameters of MS were set as follows: ion source temperature, 550°C (+) and 550°C (−); ion spray voltage, 5,500 V (+) and 4,500 V (−); curtain gas, 35 PSI; declustering potential, 100 V (+) and −100 V (−); collision energy, 10 eV (+) and −10 eV (−); interface heater temperature, 550°C (+) and 600°C (−).

### Quantification of Targeted Proteins With Liquid Chromatography–Parallel Reaction Monitoring–Mass Spectrometry (LC-PRM-MS) Analysis

The candidate protein expressions were quantified by LC-PRM-MS analysis. Three fat samples from the FY and MY groups were selected for the protein extraction, respectively. Frozen fat samples were lysed with 300 μl lysis buffer supplemented with 1 mM phenylmethanesulfonyl fluoride, followed by sonication, and centrifuged at 14,000 r/min for twice. The protein concentration was determined by BCA assay, and 10 μg proteins in each sample was separated using 12% SDS-PAGE gel. An AQUA stable isotope peptide was spiked in each sample as the internal standard reference. Tryptic peptides were loaded on C_18_ stage tips for desalting prior to reversed-phase chromatography on an EASY-nLC^TM^ 1200 System. One-hour liquid chromatography gradients, with acetonitrile ranging from 5 to 35% in 45 min, were used. LC-PRM-MS analysis was performed on a Q Exactive^TM^ Plus Hybrid Quadrupole-Orbitrap^TM^ Mass Spectrometer.

### Quantitative Reverse-Transcription PCR (qPCR) Analysis

After a series of analysis of RNA sequencing, three subcutaneous fats in each group were used for qPCR analysis. Each reverse transcription reaction consisted of 0.5 μg RNA, 2 μl of 5 × TransScript All-in-One SuperMix for qPCR and 0.5 μl of gDNA remover, in a total volume of 10 μl. Reactions were performed in a GeneAmp^®^ PCR System 9700 (Applied Biosystems, United States) for 15 min at 42°C, 5 s at 85°C. The 10-μl RT reaction mix was diluted × 10 in nuclease-free water and held at −20°C. Real-time PCR was performed using LightCycler^®^ 480 II Real-time PCR Instrument (Roche, Basel, Switzerland) with 10 μl PCR reaction mixture that included 1 μl of cDNA, 5 μl of 2 × PerfectStart^TM^ Green qPCR SuperMix, 0.2 μl of forward primer, 0.2 μl of reverse primer, and 3.6 μl of nuclease-free water. Reactions were incubated in a 384-well optical plate (Roche, Switzerland) at 94°C for 30 s, followed by 45 cycles of 94°C for 5 s and 60°C for 30 s.

### Gas Chromatography (GC) Analysis

To confirm the metabolic changes observed in non-targeted metabolomics analyses, the absolute concentrations of eicosadienoic acid (EPA), alpha-linolenic acid (ALA), and gamma-linolenic (GLA) acid were investigated according to the method described in Folch et al. (1957) and Song et al. (2017). Ten subcutaneous fat samples in the FY and MY groups were used. Fifty milligrams of subcutaneous fat was dissolved in the solution of acetone and methanol (2:1, v:v) and cleaned with anion exchange resin, and then free fatty acids were derived with boron fluoride–methanol solution. At last, the derivatives fatty acid methyl esters were determined with Agilent 7890A GC (Santa Clara, CA, United States) coupled with a flame ionization detector. The analytes were determined based on their retention times, and the fatty acid concentrations were calculated by fatty acid methyl esters.

### Statistical Analyses

Fat content, BFR, subcutaneous fat thickness, and metabolite and enzyme concentrations were analyzed using the independent-sample *T* test in SPSS 16.0, and *p* < 0.05 was considered statistically significant. The correlations between important DEG and DMs were calculated separately using Pearson correlation analysis in SPSS 16.0 as well. Raw data in transcriptomic analysis were processed using Trimmomatic, and the clean reads were mapped to the reference genome using hisat2. Meanwhile, the Q30 and GC contents of clean reads were calculated. The Fragments Per Kilobase per Million (FPKM) value of each gene was calculated using cufflinks, and the read counts of each gene were obtained by htseq-count. In this study, *p* < 0.05 and FC > 2 or FC < 0.5 were set as the threshold for significantly differential expression. DEGs were identified using the DESeq (2012) R package functions estimate SizeFactors and nbinomTest. The raw data of MS were preprocessed using the software Progenesis QI v2.3 (Nonlinear Dynamics, Newcastle upon Tyne, United Kingdom). The combined data was dealt with the R ropls package. The metabolites with variable importance in projection (VIP > 1.0 and *p* < 0.05) were screened. Principal component analysis (PCA), orthogonal partial least square discrimination analysis (OPLS-DA), GO enrichment, and KEGG pathway enrichment analysis were performed using R as well. The raw data in PRM and qPCR analysis were analyzed using Skyline 3.5.0 software (MacCoss Lab, University of Washington) and the 2^–ΔΔCt^ method (Livak and Schmittgen, 2001), respectively.

## Results

### Fat Amount in FYs and MYs

Fat amounts in FYs and MYs are shown in Table 1. The BFRs of perirenal fat, omental fat, and mesentery fat in FYs were higher than those in MYs (*p* < 0.01). The FY subcutaneous fats in the back and waist were thicker (*p* < 0.01). The LD and liver fat contents in FYs were higher (*p* < 0.01).

**TABLE 1:** Fat amount in female yaks (FYs, *n* = 10) and male yaks (MYs, *n* = 10).

**Fat amount**		**FYs (mean ± SD)**	**MYs (mean ± SD)**
BFR (%)	Perirenal fat	2.15 ± 0.17^A^	1.38 ± 0.10^B^
	Fat around liver	0.12 ± 0.01	0.12 ± 0.01
	Omental fat	2.30 ± 0.13^A^	1.35 ± 0.07^B^
	Mesentery fat	2.33 ± 0.17^A^	1.33 ± 0.06^B^
Subcutaneous fat thickness (mm)	Back	12.34 ± 1.41^A^	8.16 ± 0.45^B^
	Waist	13.78 ± 1.38^A^	12.14 ± 0.86^B^
Content (%)	Liver	2.62 ± 0.11^A^	2.21 ± 0.32^A^
	Longissimus dorsi	2.50 ± 0.08^A^	1.84 ± 0.07^B^

*Values in the same row with different capital superscripts show *p* < 0.01.*

### Concentrations of Metabolites in Serum and Enzymes in Liver

The metabolite and enzyme concentrations are shown in Table 2. HDL, LDL, CH, TG, VLDL, LEP, and COR in FY serum were higher than those in MY serum (*p* < 0.05), whereas IGF-1 was lower (*p* < 0.01). On the other hand, the FAS, ACC, SCD, DGAT-1, and CPT-1 concentrations in FY livers were higher (*p* < 0.05).

**TABLE 2:** Metabolite concentration in serum and enzyme concentration in livers of FYs (*n* = 10) and MYs (*n* = 10).

**Item**		**FYs (mean ± SD)**	**MYs (mean ± SD)**
Serum	HDL (mmol/l)	1.92 ± 0.09^A^	1.77 ± 0.06^B^
	LDL (mmol/l)	0.83 ± 0.13^A^	0.65 ± 0.03^B^
	CH (mmol/l)	2.98 ± 0.15^A^	2.66 ± 0.06^B^
	TG (mmol/l)	0.29 ± 0.02^a^	0.25 ± 0.01^b^
	NEFA (mmol/l)	0.18 ± 0.00	0.18 ± 0.00
	GLU (mmol/L)	4.68 ± 0.25	4.93 ± 0.10
	VLDL (mmol/l)	0.28 ± 0.02^a^	0.25 ± 0.01^b^
	TP (g/l)	72.17 ± 1.34	73.63 ± 2.24
	ALB (g/l)	29.53 ± 2.52	31.23 ± 1.77
	LEP (μg/l)	0.92 ± 0.05^A^	0.75 ± 0.04^B^
	COR (μg/l)	24.17 ± 1.48^a^	20.68 ± 1.70^b^
	IGF-1 (μg/ml)	0.28 ± 0.02^A^	0.34 ± 0.04^B^
	ALT (U/l)	36.45 ± 2.05	35.69 ± 1.25
	AST (U/l)	81.45 ± 4.37	80.16 ± 3.95
Liver	FAS (IU/g tissue)	6.90 ± 0.17^A^	6.15 ± 0.15^B^
	ACC (IU/g tissue)	2.40 ± 0.12^A^	2.17 ± 0.12^B^
	SCD (IU/g tissue)	6.58 ± 0.15^A^	5.99 ± 0.20^B^
	DGAT-1 (IU/g tissue)	6.17 ± 0.15^A^	5.82 ± 0.14^B^
	HSL (IU/g tissue)	7.41 ± 0.17	7.43 ± 0.18
	LPL (IU/g tissue)	0.19 ± 0.02	0.21 ± 0.03
	ATGL (IU/g tissue)	20.68 ± 1.74	21.11 ± 1.28
	CPT-1 (IU/g tissue)	27.51 ± 1.83^a^	30.26 ± 2.35^b^

*Values in the same row with different lowercase superscripts show *p* < 0.05; different capital superscripts show *p* < 0.01.*

### Transcriptome in Subcutaneous Fat

There were 1,027 DEGs in subcutaneous fat in FYs vs. MYs. Of these, 416 gene expressions downregulated, whereas 611 gene expressions upregulated. The volcano plot of total expression genes and hierarchical clustering analysis for the transcriptome profiles are shown in Supplementary Figures 1A,B, respectively. Important DEGs involved in fat metabolism in yak subcutaneous fat are shown in Table 3.

**TABLE 3:** Important differentially expressed genes (DEGs) involved in fat metabolism in FY and MY subcutaneous fat.

**Gene**	**Description**	**FC**	***P_FDR_***
*ACAA1*	3-Ketoacyl-CoA thiolase	0.27	1.27E-15
*ACAT2*	Acetyl-CoA acetyltransferase	2.22	2.04E-7
*ACOT7*	Cytosolic acyl coenzyme A thioester hydrolase	2.27	8.62E-7
*AGPAT2*	1-Acyl-sn-glycerol-3-phosphate acyltransferase beta	2.21	1.47E-10
*CPT1C*	Carnitine O-palmitoyltransferase 1	0.46	4.31E-3
*DGAT2*	Diacylglycerol O-acyltransferase 2	12.05	2.09E-76
*DHCR24*	Delta(24)-sterol reductase	2.25	1.10E-5
*ELOVL6*	Elongation of very long chain fatty acids protein 6	2.78	1.28E-5
*GPAT3*	Glycerol-3-phosphate acyltransferase 3	2.76	1.15E-3
*HACD3*	Very-long-chain (3R)-3-hydroxyacyl-CoA dehydratase 2	2.56	5.90E-3
*HADH*	Hydroxyacyl-coenzyme A dehydrogenase	2.02	2.34E-5
*HSD17B12*	Very-long-chain 3-oxoacyl-CoA reductase	2.09	6.50E-9
*LIPA*	Lysosomal acid lipase/cholesteryl ester hydrolase	2.59	2.22E-2
*LPIN1*	Phosphatidate phosphatase LPIN1	0.34	5.91E-5
*LPL*	Lipoprotein lipase	4.76	3.65E-9
*Cyp46a1*	Cholesterol 24-hydroxylase	3.26	0.11
*CYP7B1*	25-hydroxycholesterol 7-alpha-hydroxylase	2.16	0.07
*SCP2*	Non-specific lipid-transfer protein	2.54	0.04
*SCD*	Acyl-CoA desaturase	14.56	0.01
*LEP*	Leptin	29.59	7.41E-16
*SREBF1*	Sterol regulatory element-binding protein 1	2.32	3.20E-3
*APOE*	Apolipoprotein E	0.39	4.64E-14
*SLC27A4*	Long-chain fatty acid transport protein 4	2.57	2.55E-2
*LDLR*	Low-density lipoprotein receptor	32.38	0.11
*PCYOX1*	Prenylcysteine oxidase	2.26	0.03
*ADIPOR2*	Adiponectin receptor protein 2	4.11	6.37E-38
*ME1*	NADP-dependent malic enzyme	2.58	5.33E-6
*DBI*	Acyl-CoA-binding protein	3.78	1.23E-20

**FIGURE 1:**
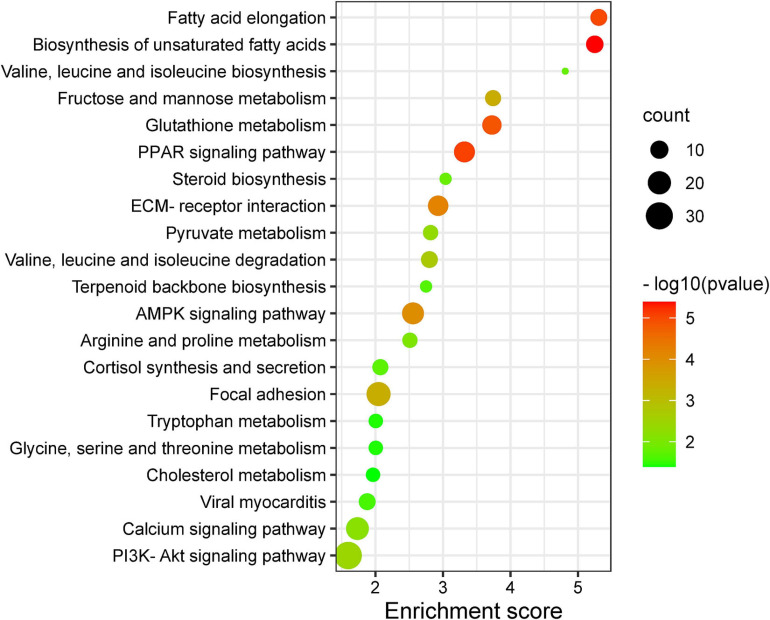
Important Kyoto Encyclopedia of Genes and Genomes (KEGG) pathway terms (*p* < 0.05) enriched by differentially expressed genes (DEGs) in subcutaneous fat of female (FYs) and male yaks (MYs). The *X*-axis means rich factor. The *Y*-axis represents the KEGG pathway terms. The roundness color represents the *p* value. The roundness area represents the DEG number in this pathway.

The GO and KEGG enrichment analyses of DEGs are shown in Supplementary Tables 1, 2, respectively. In GO analyses, the biological process mainly included biological adhesion, biological regulation, cell killing, cellular component organization or biogenesis, cellular process, and developmental process. The DEGs were enriched in 66 KEGG pathways (*p* < 0.05), and crucial KEGG pathways involved in fat metabolism are shown in Figure 1, mainly including fatty acid elongation, biosynthesis of unsaturated fatty acids, ECM–receptor interaction, focal adhesion, calcium signaling pathway, and PPAR signaling pathway. Most of above KEGG pathways were categorized as fatty acid, amino acid, and carbohydrate metabolism.

### Metabolome in Subcutaneous Fat

Unsupervised multivariate analyses were applied to provide an initial evaluation of metabolic perturbations which had been caused by gender in yak subcutaneous fat. PCA score plots showed the distribution between FY and MY subcutaneous fat as well as quality control. The quality control samples were congregated tightly in a small area, which indicated that UHPLC-TOP-MS was stable and the analysis results were reliable. Further, OPLS-DA are shown in Supplementary Figure 2A. The *R*^2^ and *Q*^2^ values in permutation testing using 200 random were 0.923 and −0.497, respectively, which showed that the model was effective and stable. There were distinct differences between the FY and MY groups, demonstrating that gender differences induced the marked perturbation of metabolites in yak subcutaneous fat. Volcano plots of metabolites in subcutaneous fat in FY vs. MY are shown in Supplementary Figure 2B, and a total of 237 DMs were obtained. Of these, 161 DM concentrations increased in FY subcutaneous fat, and 76 DM concentrations decreased. The important DMs involved in fat metabolism in yak subcutaneous fat are shown in Table 4.

**TABLE 4:** Important differential metabolites (DMs) involved in fat metabolism in FY and MY subcutaneous fat.

**Metabolites**	**FC**	**VIP**	**Score**	***P_FDR_***
Malic acid	3.59	1.45	51.8	2.02E-3
Citric acid	4.83	1.99	41.1	0.11
L-Glutamate	1.59	1.68	49.4	0.10
Glycocholic acid	8.73	4.38	56	0.02
Chenodeoxycholic acid glycine conjugate	8.80	1.61	46.8	0.02
9(S)-HpODE	2.65	1.21	53.4	0.02
13(S)-HODE	1.62	1.19	47.8	0.03
PC(15:0/14:0)	12.62	1.12	37.7	0.11
myo-Inositol	1.88	3.87	39.1	0.03
ALA	1.92	3.10	43.6	0.11
EPA	2.04	1.63	45.7	0.07
GLA	1.82	4.83	40	0.11
7-Dehydrodesmosterol	30.08	2.20	49.1	0.11
COR	3.49	1.75	38.3	0.09
D-2,3-Dihydroxypropanoic acid	1.54	1.62	38.6	0.01
2-trans,6-trans-Farnesal	0.73	2.54	37.3	0.04
Thromboxane	0.30	2.04	40.5	0.10
(±)14,15-DiHETrE	3.06	1.15	40	0.01
DPA	1.95	1.43	41.9	0.07
Calcidiol	0.19	3.53	45.2	9.20E-3

**FIGURE 2:**
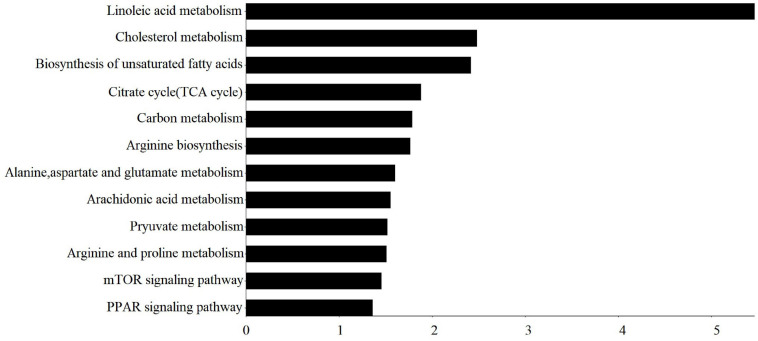
Important KEGG pathway terms (*p* < 0.05) enriched by differential metabolites (DMs) in subcutaneous fat of FYs and MYs. The *X*-axis means –log_10_*P.* The *Y*-axis represents the KEGG pathway terms.

Differential metabolites were enriched in 27 KEGG pathways (*p* < 0.05) (Supplementary Table 3), and these important KEGG pathways involved in fat metabolism are shown in Figure 2, mainly including linoleic acid metabolism, cholesterol metabolism, biosynthesis of unsaturated fatty acids, tricarboxylic acid (TCA) cycle, carbon metabolism, mammalian target of rapamycin (mTOR) signaling pathway, and PPAR signaling pathway.

### Correlations Between Important DEG and DMs

Significant correlations between relative expression levels of important DEGs and the relative concentration of important DMs in FYs and MY adipose tissue are shown in Table 5. *ELOVL6*, SCD, *LPL*, *LEP*, *APOE*, *SLC27A4*, *ADIPOR2*, *ME1*, and *DBI* gene expressions were positively correlated with ALA, EPA, GLA, and DPA concentrations. Expressions of 13 genes were negatively correlated with the calcidiol concentrations. The expressions of *LIPA* and *SLC27A4* gene were negatively correlated with the inosine concentration. *LEP* and *ME1* gene expressions were positively correlated with the COR concentration. The 13(S)-HODE concentration was positively correlated with *ELOVL6*, *HADH*, *SCD*, *LPL*, *LEP*, *SLC27A4*, *ADIPOR2*, *ME1*, and *DBI* gene expression, but negatively with *APOE* and *CPT1C* gene expressions.

**TABLE 5:** Pearson correlation of the important DEG expression involved in fat metabolism and the important DMs concentration.

**Variable**	**13(S)-HODE**	**COR**	**Inosine**	**myo-Inositol**	**ALA**	**EPA**	**GLA**	**DPA**	**Calcidiol**
*ACAA1*	−0.70	−0.52	0.64	−0.27	−0.77	−0.72	−0.74	−0.83*	0.84*
*ACOT7*	0.58	0.36	−0.61	0.11	0.63	0.60	0.61	0.70	−0.80
*CPT1C*	−0.81*	−0.58	0.68	−0.37	−0.88*	−0.65	−0.78	−0.78	0.82*
*ELOVL6*	0.81*	0.77	−0.55	0.60	0.88*	0.86*	0.90*	0.94**	−0.76
*HACD2*	0.77	0.49	−0.70	0.26	0.82*	0.56	0.71	0.71	−0.83*
*HADH*	0.83*	0.61	−0.69	0.39	0.89*	0.69	0.81	0.82*	−0.85*
*SCD*	0.83*	0.63	−0.70	0.40	0.89*	0.76	0.83*	0.87*	−0.88*
*LIPA*	0.76	0.41	−0.94**	0.13	0.74	0.56	0.62	0.58	−0.93**
*LPIN1*	−0.67	−0.61	0.49	−0.42	−0.74	−0.79	−0.78	−0.88*	0.73
*LPL*	0.89*	0.76	−0.69	0.56	0.94**	0.84*	0.91*	0.92**	−0.86*
*HSD17B12*	0.64	0.34	−0.60	0.12	0.70	0.41	0.57	0.58	−0.74
*LEP*	0.88*	0.82*	−0.69	0.62	0.91*	0.95**	0.94**	0.97**	−0.85*
*APOE*	−0.83*	−0.63	0.70	−0.40	−0.90*	−0.74	−0.83*	−0.86*	0.86*
*SLC27A4*	0.87*	0.66	−0.84*	0.40	0.88*	0.82*	0.84*	0.86*	−0.94**
*ADIPOR2*	0.82*	0.67	−0.73	0.43	0.86*	0.84*	0.86*	0.90*	−0.90*
*ME1*	0.96**	0.86*	−0.80	0.66	0.97**	0.94**	0.97**	0.95**	−0.90*
*DBI*	0.89*	0.77	−0.77	0.55	0.91*	0.92**	0.92**	0.94**	−0.90*

*p < 0.05 and **p < 0.01.

### PRM Quantification of Candidate Proteins

**FIGURE 3:**
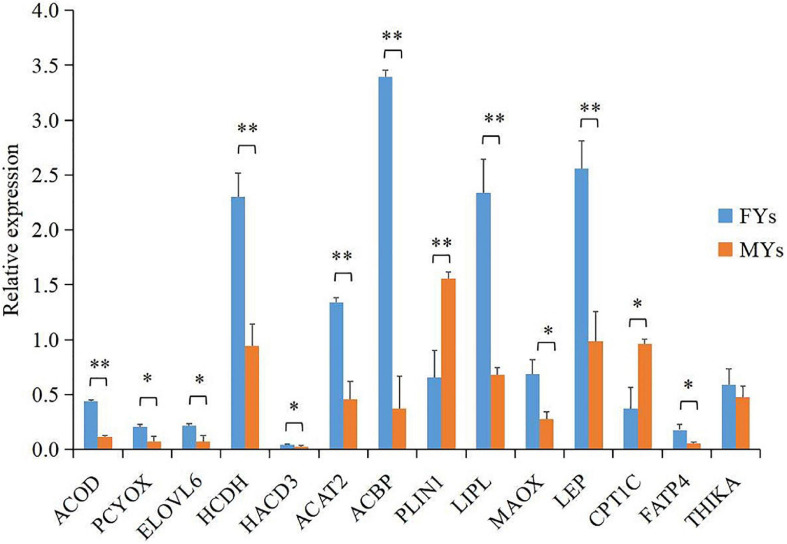
Parallel reaction monitoring (PRM) quantification of 14 proteins encoded by important DEGs in PPAR signaling. Asterisks indicate significant differences in proteins’ relative expression in subcutaneous fat between the FY and MY groups. ^∗^*p* < 0.05 and ^∗∗^*p* < 0.01.

A total of 14 proteins (Supplementary Table 4) encoded by the important DEGs were quantified by PRM, and the results (Figure 3) showed that LIPL, NADP-dependent malic enzyme (MAOX), acyl-CoA-binding protein (ACBF), hydroxyacyl-coenzyme A dehydrogenase (HCDH), elongation of very long chain fatty acid protein 6 (ELOV6), acyl-CoA desaturase (ACOD), very-long-chain (3R)-3-hydroxyacyl-CoA dehydratase 3 (HACD3), prenylcysteine oxidase 1 (PCYOX), acetyl-CoA acetyltransferase (ACAT2), hydroxyacyl-coenzyme A dehydrogenase (HADH), LEP, and long-chain fatty acid transport protein 4 (FAT4) notably increased in FY subcutaneous fat (*p* < 0.05), whereas CPT1 and perilipin-1 (PLIN1) decreased (*p* < 0.05), which generally concurred with the expression trend of DEGs which encoded the above proteins.

### Verification of Sequencing Data Using qRT-PCR

The primer details of 10 candidate genes are presented in Supplementary Table 5. Previous research indicated that the expressions of β*-actin* gene in different yak tissues are partially variable. However, a good linear relationship between the β*-actin* gene expression and the total RNA amount in the specific tissues, especially adipose tissue, was obtained in the confirmatory experiments. Therefore, the β*-actin* gene was selected as a reference gene for qPCR in yak subcutaneous fat in this study. As shown in Supplementary Figure 3A, all 10 DEGs possessed the similar expression patterns in comparison to the RNA-Seq data, indicating the reliability of our RNA-Seq data.

### Verification of DMs

The absolute concentrations of ALA, GLA, and EPA are shown in Supplementary Figure 3B, and their absolute concentrations in FY subcutaneous fat were higher those in MY subcutaneous fat (*p* < 0.05). Meanwhile, their relative concentration in FY subcutaneous fat showed significant increase in metabolomics analyses. The trends in the absolute concentration variations for three DMs in FY and MY subcutaneous fat were similar with the results from metabolomics analyses, which indicated that the UHPLC-TOP-MS non-targeted metabolomics analyses were reliable and accurate in this study.

## Discussion

The TCA cycle is the key pathway of nutrient metabolism like fat metabolism. For DMs in subcutaneous fat in FYs vs. MYs, the important pathways being related to the TCA cycle were pyruvate metabolism; biosynthesis of unsaturated fatty acids; fatty acid elongation; glutathione metabolism; fructose and mannose metabolism; valine, leucine, and isoleucine degradation; and arginine and proline metabolism. The pyruvate from amino acid and carbohydrate metabolism can be converted to acetyl CoA which can enter the TCA cycle and serve as the starting point for the long-chain fatty acids and steroid syntheses (Reed and Hackert, 1990). It is inferred that carbohydrate, amino acid, and fatty acid pathway play vital roles in yak fat metabolism. Further, citric and malic acids are formed in the TCA cycle and participates in the intermediate metabolism of carbohydrate oxidation in cattle (Ladeira et al., 2016). L-Glutamate is completely oxidized by the TCA cycle in cattle (Connolly et al., 2020). Higher concentrations of the above three DMs in FY subcutaneous fat strengthened fat metabolism by participating in the TCA cycle. Moreover, the KEGG pathway showed that DEGs are mainly involved in fatty acid, carbohydrate, and amino acid metabolism. The fat metabolism in yak is the combined effect of carbohydrate, fatty acid, and amino acid by the TCA cycle.

Cholesterol strongly associates with fat metabolism especially fat transport, and many steroid hormones affecting the fat metabolism are derived from CH. CH is the ingredient of LDL, VLDL, and HDL (Fotschki et al., 2020). The CH, LDL, VLDL, and HDL concentrations in yak serum indicated that the CH synthesis in FYs increased. VLDL transports endogenous products (Sun et al., 2010). More fat and CH were transferred in FY blood. FAS (Girard et al., 1997; Hudson et al., 2020), ACC (Smith et al., 2003), SCD (Oliveira et al., 2014), and DGAT-1 (Hu et al., 2020) are the key enzymes in fat synthesis in ruminant liver; CPT1 is a rate-limiting enzyme in fatty acid oxidation (Mørkholt et al., 2020). Their concentrations in livers indicated that the fat synthesis in FY livers increased, whereas fatty acid oxidation decreased. This tendency is beneficial to the fat deposition in FYs. The phenotypic results in serum and liver indicated that there is a positive correlation between CH and TG content in yak.

Fat deposition includes the fat synthesis and formation, growth, and expansion of lipid droplets and is regulated by multigene and transcription (Alberdi et al., 2011; Hocquette et al., 2012; Mazzucco et al., 2016). Twenty-eight DEGs involved in fat deposition were found in this study. The *DGAT2* gene has been used as a genetic marker for fat deposition in cattle (Thaller et al., 2003). An increase in *DGAT2* gene expression was previously demonstrated to be associated with an increase in fatty acid in cattle (Taniguchi et al., 2008a). *SCD* gene activity is required for lipogenic activity in subcutaneous adipose (Smith et al., 2009; Wang et al., 2017). Higher *SCD* gene expression was related to the lower total saturated fatty acids (SFAs) and higher total polyunsaturated fatty acids (PUFAs) in cattle subcutaneous fat (Yang et al., 1999). ELOVL6 is a key enzyme that regulates fatty acid composition by elongating C12-16 SFAs and PUFA (Moon et al., 2001; Chai and Tarnawski, 2002; Denic and Weissman, 2007). However, there is no report on *DGAT2*, *SCD*, and *ELOVL6* genes regulating fat deposition in yak. The concentrations of PUFAs [ALA, EPA, GLA, DPA, 9(S)-HpODE, and 13(S)-HODE] increased in FY subcutaneous fat (Moallem, 2018). Meanwhile, Pearson correlation analysis indicated that *ELOVL6* and *SCD* gene expressions were positively correlated with the concentrations of ALA, EPA, GLA, and DPA in yak subcutaneous fat. Therefore, *DGAT2*, *SCD*, and *ELOVL6* gene expressions in the subcutaneous fat of FYs with higher fat amount indicate that increased fatty acid synthesis promotes the TG deposition in yak, and overexpressions of *SCD* and *ELOVL6* genes in yak adipose tissue result in PUFA accumulation. Fat deposition is regulated through increases in not only adipocyte size but also adipocyte number (Baik et al., 2014). Several genes were identified as affecting preadipocyte proliferation and differentiation, such as *FABP4* (Gregoire et al., 1998) and LPL (Goldberg, 1996). The *LPL* gene regulates the differentiation and maturation of adipose cells in cattle (Weinstock et al., 1997; Gondret et al., 2004). In this study, the *LPL* gene is associated with adipogenic in yak. Lipogenic gene expressions are regulated by transcription factors (Taniguchi et al., 2008b; Mannen, 2011; Wei et al., 2017), and PPAR is a crucial transcription factor regulating lipogenic genes in cattle (Wang et al., 2005; Tan et al., 2006). PPAR activation produces upregulation of genes implicated in fatty acid transport and fatty binding. It has been verified that the *PPARG* gene regulates lipogenic gene expression in cattle adipose tissue (Taniguchi et al., 2008a). The *PPARD* gene is involved in fat oxidation and fatty acid synthesis, and its expression (FC = 1.42, *p* < 0.05) upregulated in FY subcutaneous fat. So the regulation of PPAR to fat synthesis in yak adipose tissue may be achieved by *PPARD* gene expression.

Some CHs regulate adipocyte size and function (Lay et al., 2001). DEGs and DMs in this study were also responsible for CH metabolism. The terpenoid backbone biosynthesis supplies the precursor compounds possessing the steroid basic skeleton for steroid biosynthesis. *PCYOX1*, *ACAT*, *FNTB*, *LIPA*, *DHCR24*, and *SQLE* genes are involved in the terpenoid backbone and steroid biosynthesis processes (Nes, 2011; Luu et al., 2014), and their expressions upregulated in FY subcutaneous fat. The concentrations of products or intermediates in steroid biosynthesis, including glycocholic acid, COR, chenodeoxycholic acid glycine conjugate, calcidiol, and 7-dehydrodesmosterol, increased in FY subcutaneous fat as well. Moreover, Pearson correlation analysis indicated that *PCYOX1*, *ACAT*, *FNTB*, *LIPA*, *DHCR24*, and *SQLE* genes were negatively correlated with the calcidiol concentration in yak subcutaneous fat. The fat quantity in FYs increased. Therefore, the active steroid biosynthesis in yak subcutaneous fat is associated with increased fat deposition as well. Existing studies mainly focused on the dietary effect on cholesterol synthesis (Li et al., 2015). The above results highlighted the contribution of steroid synthesis to fat deposition in yaks from the viewpoint of molecular biology.

**FIGURE 4:**
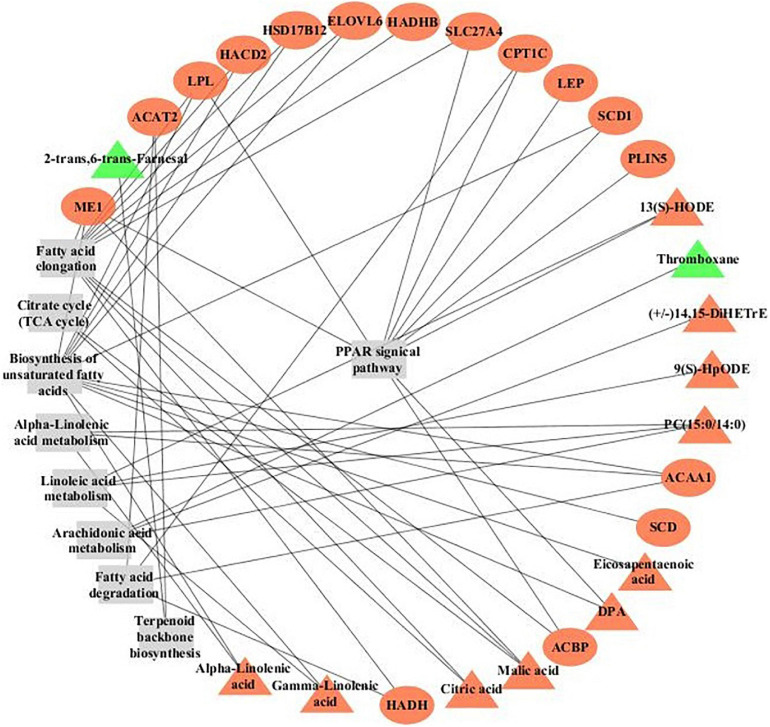
The regulatory networks of DEGs and DMs based on KEGG Markup Language (KGML) in subcutaneous fat of FYs and MYs. Triangles represent metabolites, circles represent genes, and rectangles represent pathway names. Red denotes the upregulated gene or metabolite, and green denotes the downregulated gene or metabolite.

Fat deposition is coordinately regulated through many signaling pathways that integrate fat synthesis with fat utilization (Gui et al., 2017). *ME1*, *SCD*, *DBI*, *LPL*, *CPT1*, *SLC27A4*, *ACAA1*, and *PLIN1* genes were significantly enriched in the PPAR signaling pathway, and the *LIPA*, *DHCR24*, and *SQLE* gene were significantly enriched in the steroid biosynthesis pathway. Moreover, calcium signaling pathway and junction-related pathways including focal adhesions and ECM–receptor interaction also obtained a higher enrichment score. The regulatory networks of DEGs and DMs based on KEGG Markup Language (KGML) provided a comprehensive profile of the mechanisms contributing to fat deposition in yak with different genders in Figure 4. Steroid cortisol plays a crucial role in fat metabolism (Daix et al., 2008). Adipocytokine activates transcription factors regulating fat deposition by the junction-related pathways (Weibel et al., 2012; Khor et al., 2013). It is inferred that high level hormones from exocytosis secretion in FYs, including COR and myo-inositol, active the calcium signaling in adipocyte, and then the LEP secretion is actived by calcium mediated signal transduction system (Alarcona et al., 2018). LEP concentrations are an important indicator in evaluations of fat amount in breeding programs (Bonnet et al., 2007). Growing fat depots upregulate *LEP* gene expression (Thomas et al., 2002). High concentrations of LEP in FY subcutaneous fat activated PPAR signaling by the junction-related pathways. Then, the upregulated expressions of lipogenesis genes *LPL*, *ME1*, and *SCD* promote TG synthesis in FY subcutaneous fat. Meanwhile, the upregulated expressions of *LIPA*, *DHCR24*, and *SQLE* genes increase steroid synthesis. The expressions of 14 proteins quantified by PRM concurred with the expression trend of DEGs encoding the above proteins in FY and MY subcutaneous fat, which further verified the crucial candidate gene and signaling pathways for fat deposition in yak.

## Conclusion

In this study, we have investigated the effect of gender to fat deposition in yaks. The capacity of fat deposition in FYs was stronger than that in MYs, and the fat amounts in FYs were higher. The fat and cholesterol synthesis in FY liver and the fat transport in FY blood increased. The transcriptomic and metabolomic analyses indicated that fat metabolism in yak is the combined effect of carbohydrate, fatty acid, and amino acid metabolism. The increase of TG metabolism in FY subcutaneous fat was achieved by the increase of the steroid synthesis and *ME1*, *SCD*, *DBI*, *LPL*, *CPT1*, *PLIN1*, *LIPA*, *DHCR24*, and *SQLE* gene expression. PPAR signaling and steroid biosynthesis pathway play a crucial role in this process. In FY subcutaneous fat, higher-level COR and myo-inositol activate the calcium signaling in adipocytes, followed by the activation of LEP secretion by the calcium-mediated signal transduction system, and PPAR signaling was activated by the junction-related pathways at last.

## Data Availability Statement

The datasets generated for this study can be found in the Sequence Read Archive (https://www.ncbi.nlm.nih.gov/sra) at NCBI, with the BioProject ID: PRJNA686190.

## Ethics Statement

The animal study was reviewed and approved by the Ethics Committee of the Lanzhou Institute of Husbandry and Pharmaceutical Sciences, Chinese Academy of Agricultural Sciences. Written informed consent was obtained from the owners for the participation of their animals in this study.

## Author Contributions

LX: conceptualization and writing – review and editing. JP: methodology. XW and QK: validation. XG: writing – review and editing. PY: conceptualization, investigation, and writing – review and editing. All authors contributed to the article and approved the submitted version.

## Conflict of Interest

The authors declare that the research was conducted in the absence of any commercial or financial relationships that could be construed as a potential conflict of interest.

## Publisher’s Note

All claims expressed in this article are solely those of the authors and do not necessarily represent those of their affiliated organizations, or those of the publisher, the editors and the reviewers. Any product that may be evaluated in this article, or claim that may be made by its manufacturer, is not guaranteed or endorsed by the publisher.

## Abbreviations

BFR: body fat rate
COR: cortisol
PPAR: peroxisome proliferators-activated receptor
RNA-Seq: RNA-sequencing
MS: mass spectrum
DEGs: differentially expressed genes
DMs: differentially metabolites
GO: gene ontology
KEGG: Kyoto Encyclopedia of Genes and Genome
LC-PRM-MS: liquid chromatography–parallel reaction monitoring–mass spectrometry
GC: gas chromatography
LD: longissimus dorsi
GLC: glucose
CH: cholesterol
TG: triglyceride
HDL: high-density lipoprotein cholesterol
LDL: low-density lipoprotein cholesterol
NEFA: non-esterified fatty acid
TP: total protein
ALB: albumin
VLDL: very low-density lipoprotein
LEP: leptin
IGF-1: insulin-like growth factor-1
FAS: fatty acid synthase
ACC: acetyl-CoA carboxylase
SCD: stearoyl-CoA desaturase
DGAT-1: diacylglycerol acyltransferase 1
HSL: hormone-sensitive lipase
LPL: lipoprotein lipase
ATGL: adipose triglyceride lipase
CPT-1: carnitine palmitoyl transferase 1
ALT: alanine aminotransferase
AST: aspartate aminotransferase
ELISA: enzyme-linked immunosorbent assay
qPCR: quantitative reverse-transcription PCR.
