# Supplementary material for: Effect of Gender to Fat Deposition in Yaks Based on Transcriptomic and Metabolomics Analysis
Source: Front Cell Dev Biol. 2021 Aug 24;9:653188. doi: 10.3389/fcell.2021.653188 (PMC8421605; doi:10.3389/fcell.2021.653188)
Supplement: Supplementary file 1 [file Data_Sheet_1.DOC]

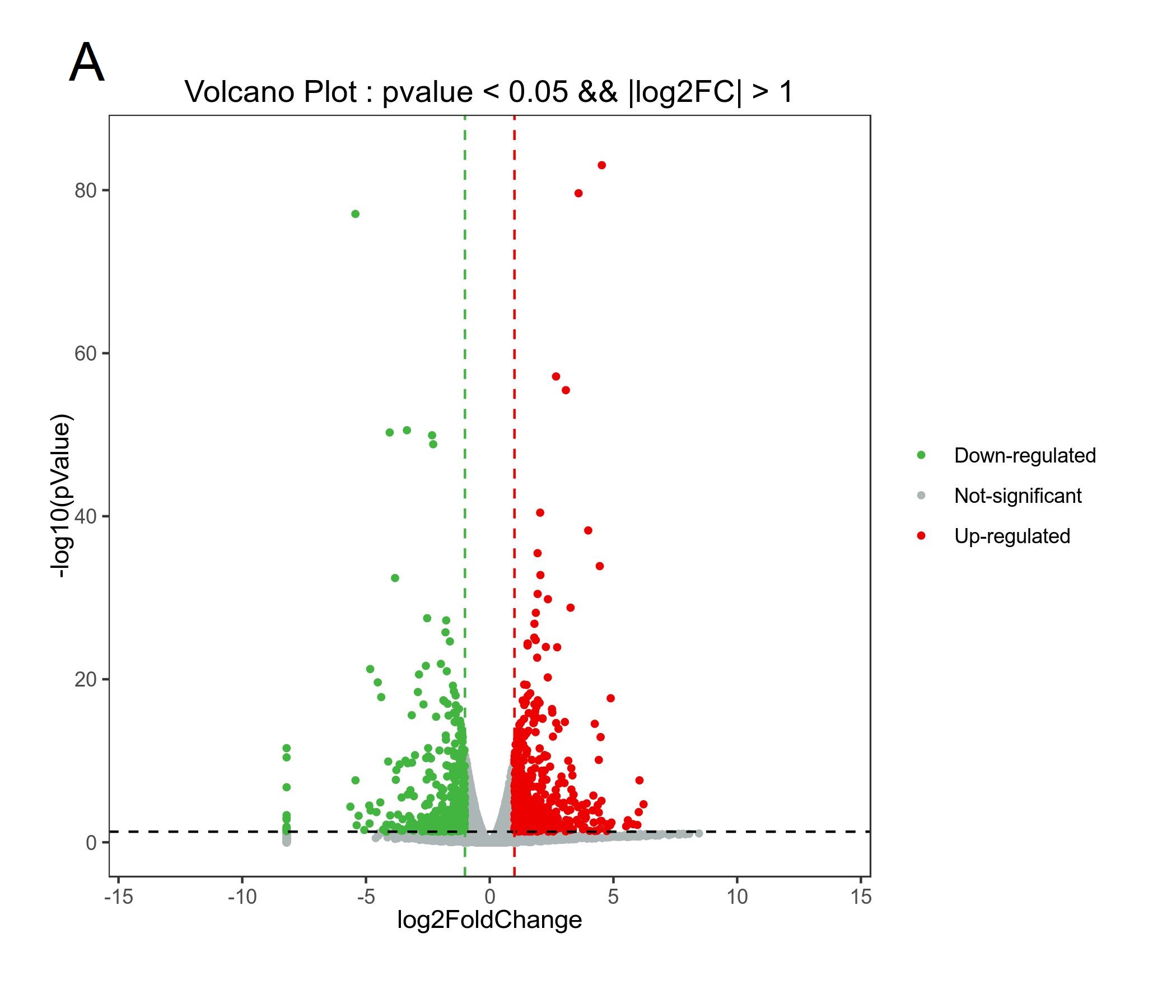

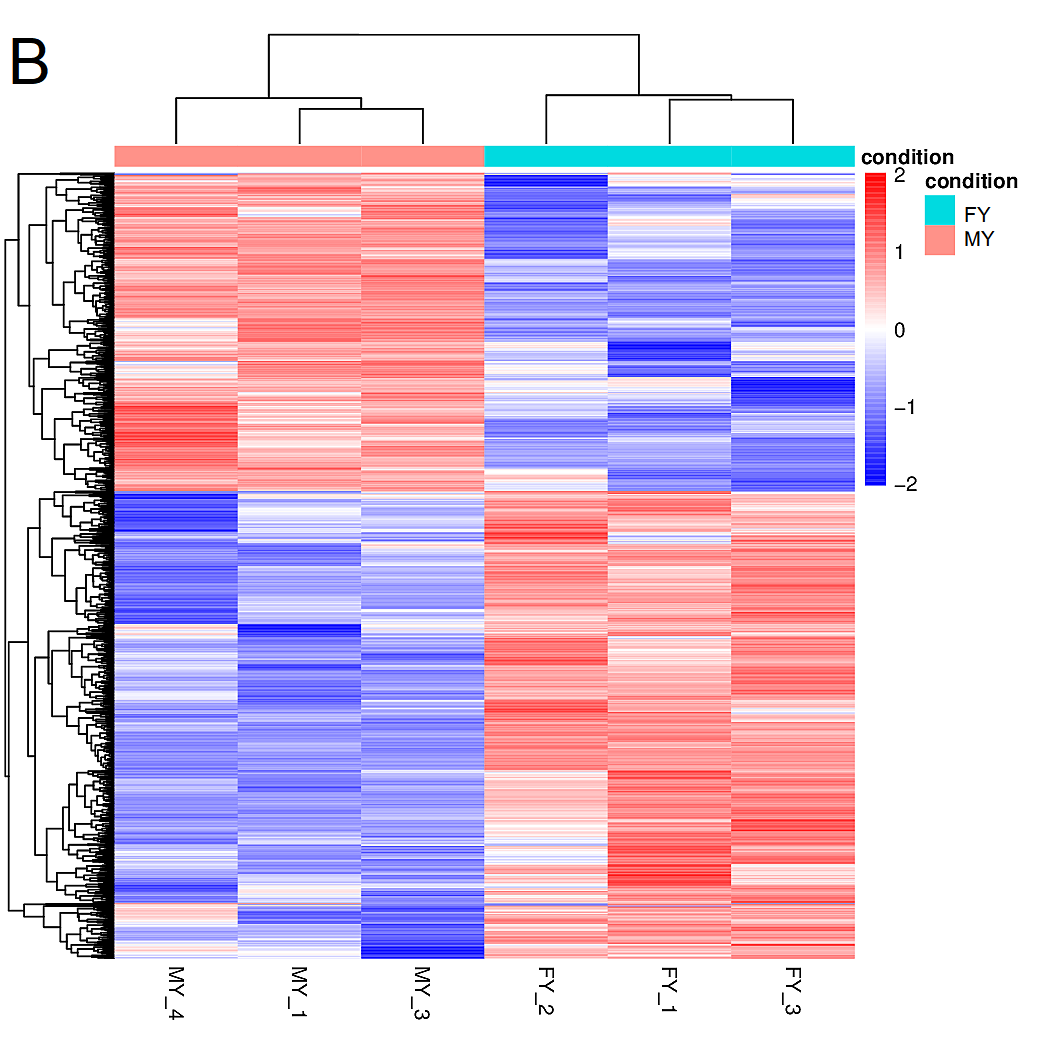


Supplementary Figure 1 A The volcano plot of total expression genes in subcutaneous fat of

male and female yaks; Red dots indicate upregulated differentially expressed genes (DEGs),

green dots indicate downregulated DEGs and grey dots indicated non-differentially

expressed genes. B Clustering of the DEGs in the subcutaneous of male and female

yaks. Columns indicate individual samples, rows represent each DEG, and the color

scale represents the relative expression level of the DEGs.
