# Supplementary material for: Effect of Gender to Fat Deposition in Yaks Based on Transcriptomic and Metabolomics Analysis
Source: Front Cell Dev Biol. 2021 Aug 24;9:653188. doi: 10.3389/fcell.2021.653188 (PMC8421605; doi:10.3389/fcell.2021.653188)
Supplement: Supplementary file 2 [file Data_Sheet_2.DOC]

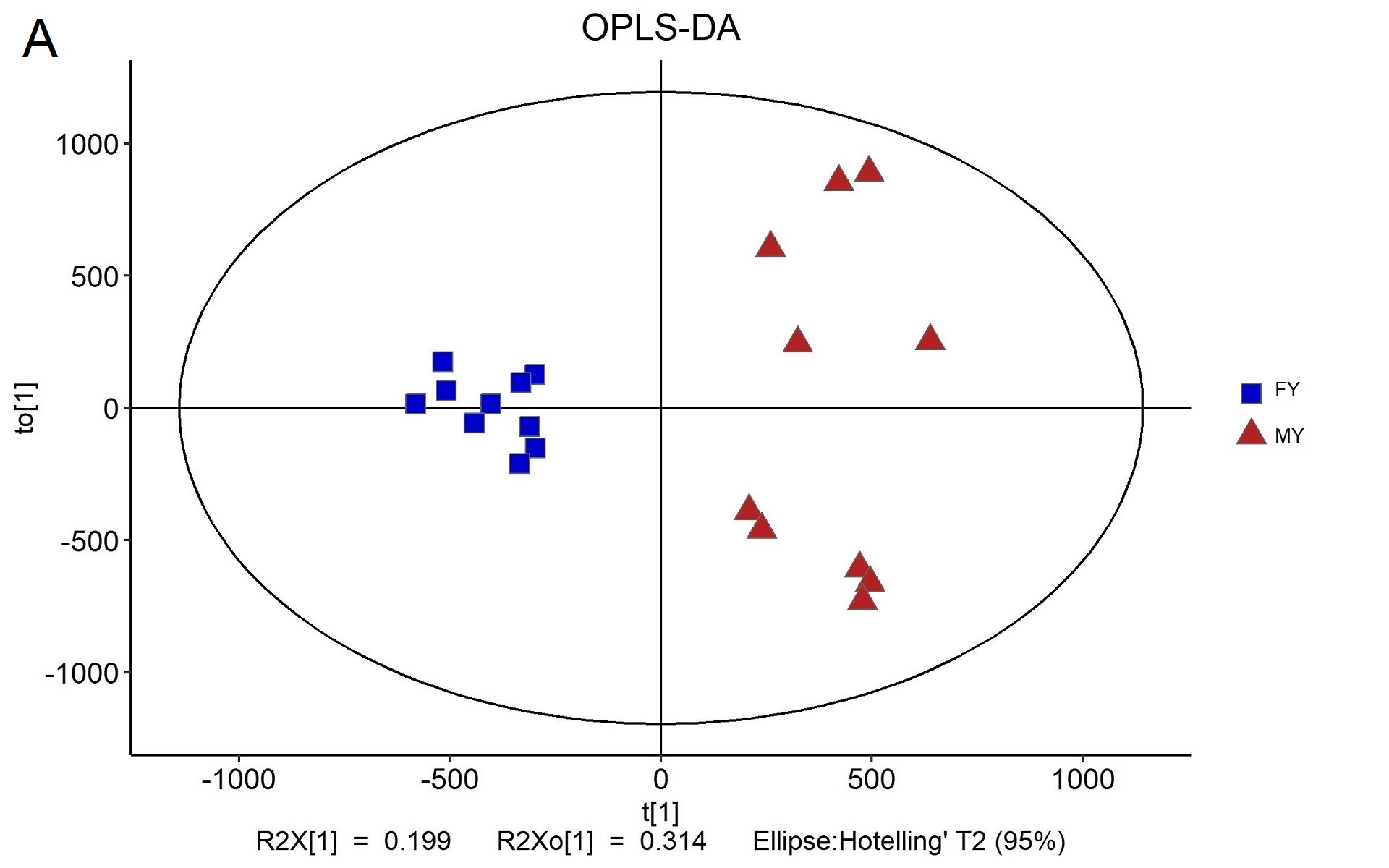


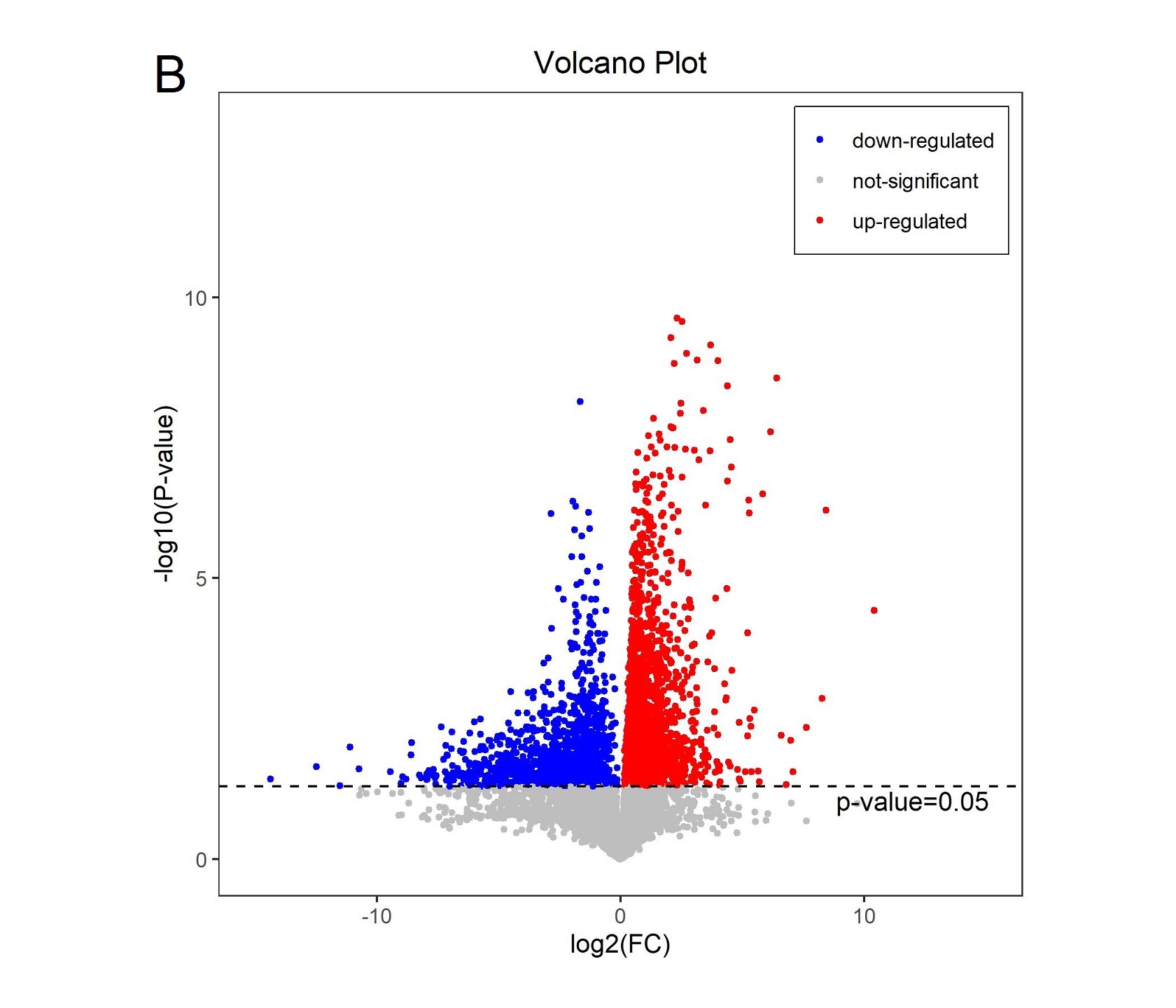


Supplementary Figure 2 A Orthogonal partial least squares discrimination analysis (OPLS-DA) of metebolites in subcutaneous fat of famale yaks and male yaks. B Volcano plot of metebolites in subcutaneous fat of famale yak compared with male yak.
