# Supplementary material for: Effect of Gender to Fat Deposition in Yaks Based on Transcriptomic and Metabolomics Analysis
Source: Front Cell Dev Biol. 2021 Aug 24;9:653188. doi: 10.3389/fcell.2021.653188 (PMC8421605; doi:10.3389/fcell.2021.653188)
Supplement: Supplementary file 3 [file Data_Sheet_3.doc]

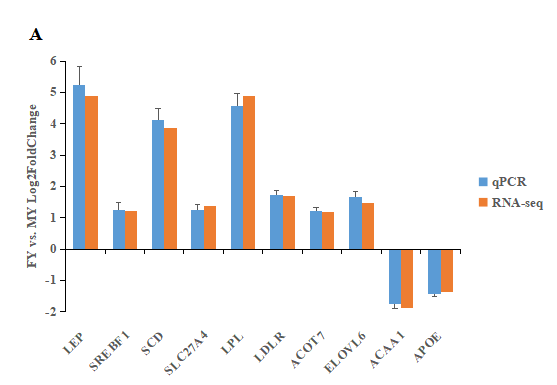


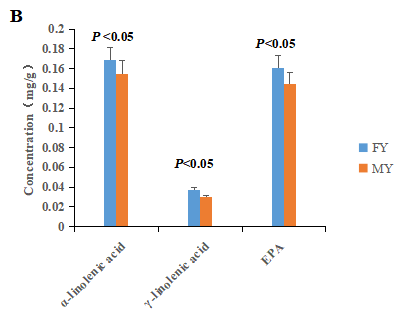


Supplementary Figure 3 A Validation and comparison of log_2_ (fold change) in ten DEGs between qRT-PCR and RNA-Seq. B The absolute concentration of α-linolenic acid, γ-linolenic acid and EPA in subcutaneous fat of FYs and MYs by the GC analysis. FY, female yaks; MY, male yaks.
