# Supplementary material for: Effect of Gender to Fat Deposition in Yaks Based on Transcriptomic and Metabolomics Analysis
Source: Front Cell Dev Biol. 2021 Aug 24;9:653188. doi: 10.3389/fcell.2021.653188 (PMC8421605; doi:10.3389/fcell.2021.653188)
Supplement: Supplementary file 7 [file Data_Sheet_7.docx]

Supplemental Table 4 The informations of 14 proteins encoded by important DEGs in subcutaneous fat of FYs and MYs

| Protein accession | Protein description | Protein symbol | Gene  name |
| --- | --- | --- | --- |
| XP_014335786.1 | Lipoprotein lipase | LIPL | *LPL* |
| XP_005903380.1 | NADP-dependent malic enzyme | MAOX | *ME1* |
| XP_005910864.1 | Acyl-CoA-binding protein | ACBP | *DBI* |
| XP_005902329.1 | Perilipin-2 | PLIN2 | *PLIN2* |
| XP_005892117.1 | Acyl-CoA desaturase | ACOD | *SCD* |
| XP_005900036.1 | Very-long-chain(3R)-3-hydroxyacyl-CoA dehydratase 3 | HACD3 | *HACD3* |
| XP_005893664.1 | Prenylcysteine oxidase 1 | PCYOX | *PCYOX1* |
| XP_005896285.1 | Elongation of very long chain fatty acids protein 6 | ELOV6 | *ELOVL6* |
| XP_005900453.1 | Acetyl-CoA acetyltransferase | ACAT2 | *ACAT2* |
| XP_005896308.1 | Hydroxyacyl-coenzyme A dehydrogenase | HCDH | *HADH* |
| XP_005908961.1 | Carnitine O-palmitoyltransferase 1 | CPT1C | *CPT1C* |
| XP_005909757.1 | Leptin | LEP | *LEP* |
| XP_005910104.1 | Long-chain fatty acid transport protein 4 | FATP4 | *SLC27A4* |
| XP_005901200.1 | 3-ketoacyl-CoA thiolase | THIKA | *ACAA1* |
